# Control techniques for creep deformation of surrounding rock in deep underground roadways

**DOI:** 10.1371/journal.pone.0326803

**Published:** 2025-11-06

**Authors:** Lijie Ge, Dong Liu, Jiaxing Tao, Zhuang Zhang, Hao Lei, Yan Zhao

**Affiliations:** 1 School of Economics and Management, Hebei Institute of Architectural Engineering, Zhangjiakou, Hebei, China; 2 Hebei Province Green Building Collaborative Innovation Center, Zhangjiakou, Hebei, China; 3 School of Mechanics and Civil Engineering, China University of Mining and Technology (Beijing), Beijing, China; 4 Glodon Company Limited), Beijing, China; China University of Mining and Technology, CHINA

## Abstract

As resource extraction extends to greater depths, surrounding rock in deep underground roadways exhibits pronounced creep deformation due to the coupled effects of high in-situ stress and time-dependent behavior. Conventional support systems face significant challenges in maintaining long-term stability under such conditions. This study focuses on the pump station roadway of a mine in North China as a case study and conducts an integrated investigation involving theoretical analysis, physical modeling, and numerical simulation to develop effective creep control strategies. A theoretical framework for creep deformation control is established based on the radial stress gradient mechanism of the surrounding rock. A composite support system—comprising concrete-filled steel tube (CFST) supports, staged grouting, rock bolts, and sprayed concrete—is proposed. Using a self-developed two-dimensional physical modeling apparatus, the deformation and stress evolution of the surrounding rock are systematically compared under unsupported and composite-supported conditions, identifying key deformation zones and dominant creep patterns. Furthermore, a three-dimensional numerical model incorporating a damage-coupled creep constitutive relationship is constructed to evaluate the support system’s effectiveness in controlling roof subsidence, sidewall convergence, and plastic zone expansion. Results demonstrate that the CFST-based composite support system significantly mitigates creep-induced instability and enhances long-term roadway stability, offering theoretical and practical guidance for the design and optimization of support systems in deep soft rock roadways.

## 1 Introduction

With the continued advancement of China’s national energy strategy, coal remains a dominant component of the energy mix. As fundamental spatial structures in coal mining operations, mine roadways play an irreplaceable role in ventilation, transportation, drainage, and disaster mitigation. In deep mining environments, roadway stability is directly linked to the safe and efficient operation of the entire mining system. The long-term performance of these underground structures has emerged as a key technical challenge that constrains the sustainable extraction of deep coal resources [1–3]. Consequently, investigating the deformation and failure mechanisms of deep mine roadways and developing effective control technologies is of significant theoretical and practical importance [4].

Compared to shallow roadways, deep underground tunnels are typically subjected to complex geological conditions, including high in-situ stress, tectonic disturbances, and extensively developed jointed rock masses. These factors make deep roadways highly susceptible to time-dependent deformation, with creep behavior being particularly pronounced [5–7]. Under long-term loading, the surrounding rock undergoes continuous slow deformation, typically following a three-stage process: initial deformation, steady-state development, and accelerated failure. This progressive deformation can undermine the stability of the support system, induce the disintegration of the surrounding rock, and, in severe cases, lead to large-scale roadway damage, repeated deformation repairs, or even partial collapse [8–11].

To address these challenges, Zhu investigated the asymmetric deformation of roadway rock masses and implemented continuous mining and backfilling technologies, achieving improved support performance. Meng et al. explored the deformation and failure mechanisms of deep roadways under dynamic loading and proposed corresponding control strategies [12]. Chen et al. examined the deformation behavior of fractured deep roadways influenced by blasting and other dynamic disturbances. Their study proposed optimized control measures, which were validated through numerical and physical modeling [13]. Wang based on a true triaxial loading system combined with AE and DIC techniques revealed the evolutionary mechanisms of roadway failure patterns under different stress deflection angles. The study highlighted that stress orientation not only directly affects the instantaneous failure morphology but also plays a crucial regulatory role in the long-term creep process by governing crack propagation paths and cumulative damage [14]. However, most existing studies focus on short-term deformation mechanisms and support responses. Comprehensive understanding of the evolution of creep behavior, the critical instability thresholds, and the failure mechanisms in deep roadways remains limited [15].

To investigate the creep development laws of deep roadway surrounding rock, scholars have conducted extensive research and achieved significant results. Wang, through laboratory experiments and a damage-bonding model, revealed that in hard–soft composite rocks (sandstone–mudstone) under high stress, mudstone exhibits much greater creep damage than sandstone, ultimately governing the failure and large deformation of roadway sidewalls. The study emphasized the necessity of reinforcing sidewall support and considering the feedback effect between the roof and sidewalls [16]. Geng, taking the deep roadways of the Huize lead–zinc mine in Yunnan as an example, examined the timing and parameter optimization of secondary combined support. The results showed that under high in situ stress, the surrounding rock exhibited pronounced rheological characteristics, and that rational timing of secondary support should be determined based on radial displacement monitoring and creep tests. Furthermore, the study optimized parameters such as shotcrete thickness, bolt length, spacing, and diameter through numerical simulations and orthogonal tests, thereby improving stability while reducing support costs [17]. Zhao et al. conducted triaxial creep–disturbance tests on coal samples and monitored microcrack propagation with acoustic emission technology. Their results demonstrated that coal strength deteriorates significantly under creep disturbance, and they proposed an improved nonlinear creep model to quantify long-term evolutionary parameters [18]. Sun et al. systematically studied the creep behavior of thawed surrounding rock by pre-embedding fissures of varying angles and positions in straight-wall semi-circular arch models and employing DIC, strain gauges, and ultrasonic monitoring. Their findings indicated that fissure geometry strongly alters the deformation patterns and damage evolution paths of the surrounding rock, resulting in complex shear–compression deformation and local stress concentrations, thereby intensifying sidewall damage and potentially inducing rockburst-like failure [19]. Ma et al. proposed a novel four-dimensional support (4D support) system, which establishes a new equilibrium structure with three-dimensional compressive confinement to effectively suppress creep instability of deep roadway surrounding rock. Numerical simulations and in situ monitoring confirmed that when the burial depth exceeds 520 m, 4D support, compared with conventional methods, better restrains the expansion of the plastic zone, reduces the rate of elastic energy accumulation, and maintains long-term stability of the surrounding rock for up to six months [20].

Extensive research has been conducted to investigate the creep behavior of deep underground roadways, yielding significant insights. Deng, using the Dapingshan Tunnel as a case study, identified creep parameters of the surrounding rock through laboratory testing and employed inverse displacement analysis to reconstruct the initial stress field under high in-situ stress conditions. This enabled a systematic exploration of the mechanisms behind large tunnel deformations due to creep [21]. Huang conducted triaxial creep tests on Jinping marble and revealed the coupled effects of pore water pressure and excavation-induced damage on rock creep deformation, strength, and failure modes. Based on experimental findings, a nonlinear viscoelastic–plastic damage (VEPD) model incorporating pore pressure effects was developed to simulate the full creep evolution of deep surrounding rock masses [22]. Tran proposed a time-dependent constitutive model based on the aging-induced degradation of rock strength and elastic modulus, and derived a semi-analytical solution for circular deep tunnels considering the advancing tunnel face [23]. Sun investigated the coupled mechanisms of creep damage and strain softening in granite through experiments, and subsequently developed a nonlinear coupling model for numerical simulations. This model accurately predicted the deformation and progressive failure behavior of deep hard rock roadways [24].

In existing studies, some researchers have attempted to analyze the creep response of tunnels using theoretical models and numerical simulations. However, the predictive capacity of these approaches remains limited due to model simplifications and uncertainties in constitutive parameters, particularly when describing the long-term coupled evolution of the surrounding rock–support system [25–28]. In particular, two-dimensional physical model tests simulating creep in deep roadways remain scarce. There is a lack of controllable and visualizable physical modeling techniques capable of accurately reconstructing the full creep process in deep underground settings. This scarcity is especially evident in the absence of 2D creep similarity model tests that can realistically capture the progressive failure features, stress redistribution patterns, and displacement–velocity evolution at critical locations such as the roof, sidewalls, and haunches of the tunnel.

In this context, this study takes the deep pump station roadway of a mine in North China as the research object and develops a self-designed two-dimensional experimental system for simulating creep control in deep tunnels. A series of similarity model tests are conducted to investigate the interaction between surrounding rock and the support system. By comparing the deformation and stress evolution under unsupported and composite-supported conditions, the dominant mechanisms governing creep-induced instability under high in-situ stress are elucidated, and the effectiveness of the proposed concrete-filled steel tube (CFST) composite support system is experimentally verified. Building upon the physical modeling results, a three-dimensional numerical model of the roadway is established. A damage-coupled creep constitutive relationship is incorporated into the model and implemented using the FLAC3D platform to simulate the long-term evolution of stress, displacement, and plastic zone development in the surrounding rock. The findings confirm the control efficacy and mechanistic reliability of the proposed support system in managing creep deformation in deep underground environments.

## 2 Project overview

**Fig 1:**
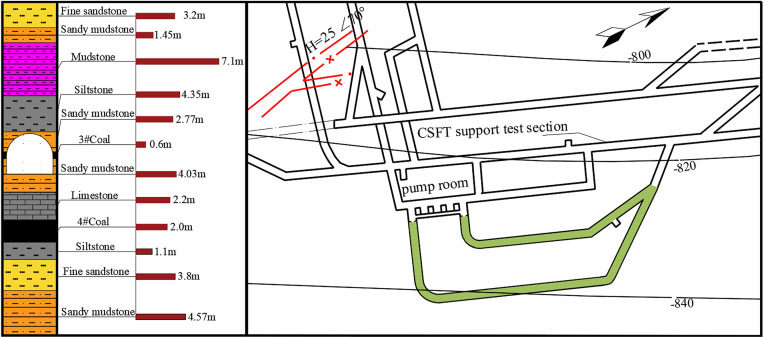
Plan layout of the roadway.

In the studied project focused on the pump station roadway of a mine in North China, the surrounding rock mass primarily consists of sandy mudstone interbedded with No. 3 coal seams. The rock mass is generally weak and exhibits low mechanical strength. The roadway is buried at a depth of 965 m and features a straight-wall semi-circular arch profile, with cross-sectional dimensions of 4.2 m × 3.2 m. According to the geological section, both sidewalls of the roadway are composed of a 0.6 m thick No. 3 coal seam, while the roof and floor are formed by sandy mudstone layers with thicknesses of 4.02 m and 4.03 m, respectively. These are further bounded by overlying and underlying strata of 3.1 m thick siltstone and 2.2 m thick limestone. The roadway layout and lithological distribution are shown in Fig 1 [25]. The complex configuration of soft and hard rock strata, coupled with high in-situ stress conditions, leads to poor stability of the surrounding rock.

**Fig 2:**
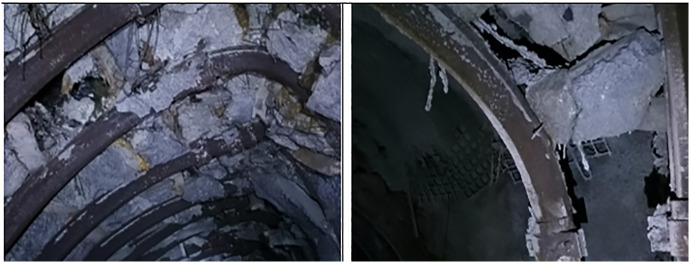
Deformation and failure characteristics of the roadway.

Field investigations revealed severe deformation and failure of the roadway during service. Significant buckling of the steel support arches was observed, accompanied by large-scale spalling of the roof rock, cracking and detachment of the shotcrete layer, and localized occurrences of roof collapse and sidewall instability. As shown in Fig 2, these phenomena highlight the inadequacy of the original support system under conditions of high in-situ stress and creep-induced deformation.

**Fig 3:**
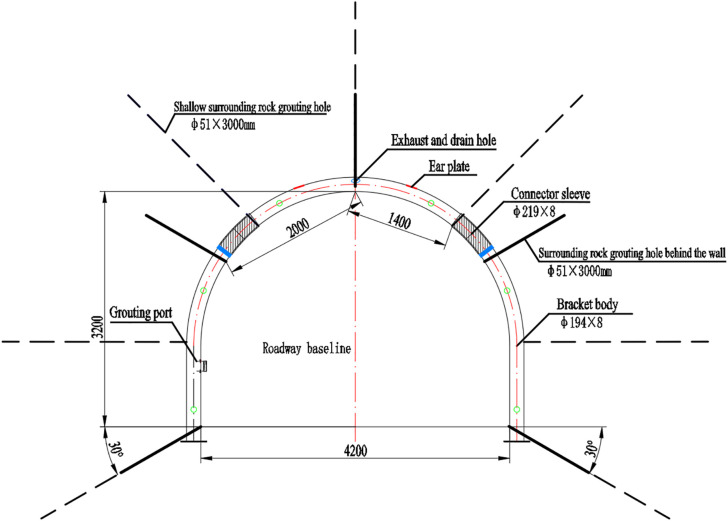
Composite support scheme based on concrete-filled steel tube (CFST) arches.

To effectively address the large-deformation challenges in deep roadways, a composite support system was implemented, emphasizing high bearing capacity and pressure equalization. The system comprises circular concrete-filled steel tube (CFST) arches, backfill and shallow rock grouting, welded steel mesh, surface shotcrete, and reinforcement rock bolts, as illustrated in Fig 3. The proposed support system consists of a circular concrete-filled steel tube (CFST) frame with a cross-sectional dimension of 4.2 m × 3.2 m. The frame adopts seamless circular steel tubes with a specification of φ194 × 8 mm, assembled from three segments. Each segment is connected using φ219 × 8 mm couplers with a length of 600 mm, while adjacent frames are installed at a spacing of 800 mm and linked by six struts. Between the frame and the surrounding rock, reinforcement mesh and timber laggings are installed. To further improve stability, the system incorporates backfilling grouting (boreholes of 1 m depth, five per row, spacing 3.2 m, designed pressure 3 MPa) and shallow grouting (boreholes of 3 m depth, seven per row, spacing 1.6 m, designed pressure 4 MPa). Together, these measures form a composite arch structure of “frame–surrounding rock–filling body,” which effectively redistributes concentrated loads and enhances the stability of the surrounding rock.

In terms of construction, the CFST frame is divided into a crown segment and two leg segments, which are installed on-site using rail-mounted forepoling beams and chain platforms to ensure precise alignment with the roadway cross-section. After frame assembly, reinforcement mesh is fixed at the rear of the structure, followed by C40 concrete grouting. The grout is then cured until the required design strength is achieved. Additionally, six φ20 × 2400 mm threaded steel resin bolts are installed symmetrically on both sidewalls to restrain lateral convergence and suppress the expansion of the plastic zone.

## 3 Creep control theory for deep underground roadways

Following excavation, the stress state of the surrounding rock undergoes significant changes, transitioning from a triaxial stress condition in the undisturbed deep zone to an approximately uniaxial state near the tunnel boundary due to stress relief. This results in a radial stress gradient from the tunnel wall into the deeper rock mass, reflecting the complex mechanical interaction between the surrounding rock and the support system during the excavation process. Under this stress gradient, the deep surrounding rock experiences continuous time-dependent creep deformation directed toward the tunnel interior. As time progresses, the extent of this deformation increases, potentially leading to support system failure and overall tunnel instability. Therefore, any investigation into roadway failure mechanisms must treat the stress state of the surrounding rock, its creep deformation, and the response of the support system as an interdependent and integrated system.

**Fig 4:**
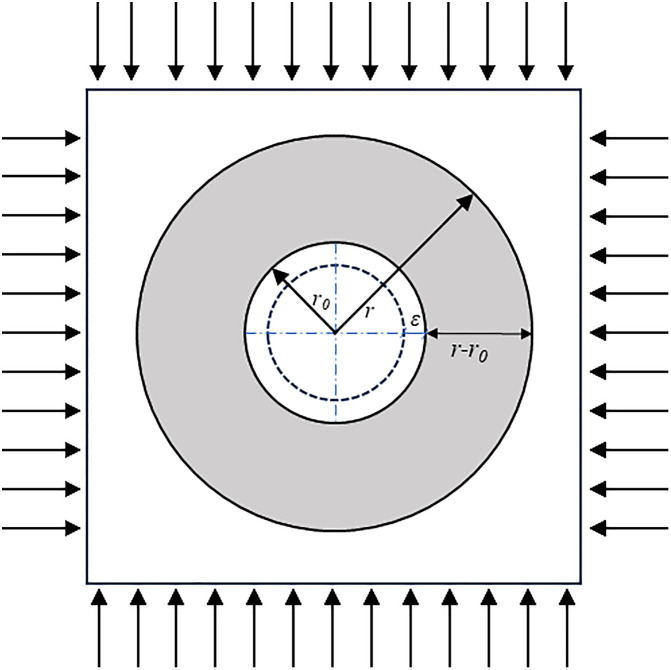
Stress distribution in the surrounding rock of the deep roadway.


As illustrated in Fig 4, consider a circular tunnel excavated in a homogeneous rock mass. After a time increment Δt, the surrounding rock exhibits a creep strain ε directed toward the tunnel interior. The relationship between the radial stress gradient and the creep rate of the surrounding rock can be expressed as [29]:

$$V=\lambda (\sigma_{r}-\sigma_{r0})/(r-r_{0})$$ (1.1)

In the above expression, *V* denotes the creep velocity of the surrounding rock, and *λ* is the creep coefficient, which is related to the intrinsic physical properties of the rock. A higher *λ* value indicates a faster creep rate. *σ*_*r*_ represents the radial stress at a distance r from the tunnel center. The equation clearly shows that the creep velocity is directly proportional to the radial stress gradient. A steeper gradient corresponds to more pronounced creep deformation within the tunnel surroundings. This formulation highlights that the radial stress gradient is one of the critical factors influencing the creep behavior of the surrounding rock. Therefore, in order to effectively control and mitigate creep deformation, it is essential to reduce the magnitude of this gradient. Based on this conclusion, two primary strategies can be adopted to reduce the radial stress gradient and thereby improve the control of time-dependent deformation in deep underground roadways.

### (1) Increasing the support reaction force at the tunnel boundary

By enhancing the support resistance at the tunnel boundary, the radial stress difference between the tunnel wall and the undisturbed deep surrounding rock can be effectively reduced. Since the stress in the deep rock mass remains close to the initial in-situ stress, increasing the support reaction near the tunnel boundary to a level comparable with the far-field stress can theoretically reduce the creep velocity *V* to zero, thereby suppressing creep deformation. This can be achieved by increasing the stiffness and strength of the support structure, thereby improving its load-bearing capacity. Through this approach, the stress differential between the tunnel wall and the deep rock can be minimized, leading to a significant reduction in the creep rate of the surrounding rock.

### (2) Optimizing pressure-relief methods and distances

By adopting appropriate pressure-relief techniques and setting a reasonable unloading distance, the peak stress zone can be shifted deeper into the surrounding rock, thereby reducing stress concentrations at the tunnel boundary. Although the stress differential between the tunnel wall and the far-field rock may remain unchanged, increasing the distance between the stress peak and the tunnel center effectively alleviates stress concentration near the excavation surface, thus mitigating the risk of creep-induced deformation. The above two theoretical approaches provide a rational framework for controlling creep deformation in deep roadway surroundings. They offer guidance for selecting appropriate support technologies in engineering design, allowing the implementation of targeted measures to reduce long-term deformation risks in deep underground tunnels.

## 4 Creep control model testing for deep underground roadways

### 4.1 Similarity test design

In the design of physical similarity models, beyond conventional similarity criteria such as geometry, stress, and modulus, ensuring the creep compatibility of similar materials is essential for the reliability of long-term deformation simulations. Previous studies have demonstrated that modified mortar, gypsum–bentonite composite systems, and polymer-admixed materials can effectively reproduce the viscoelastic characteristics of prototype rock masses [30–32].

This study takes the pump station roadway of a mine in North China as the engineering background. River sand, cement, gypsum, and water were selected as the primary similarity materials to simulate the mechanical behavior of the surrounding rock. Based on the lithological distribution characteristics of the roadway, the three-dimensional stress state of the surrounding rock was simplified as a plane strain problem for modeling purposes. A two-dimensional physical model of the roadway was constructed using the selected similarity materials. The experimental objective is to investigate the stress evolution, deformation patterns, and failure characteristics of the surrounding rock under stepped creep loading following excavation. The test also aims to characterize the time-dependent creep behavior at various positions and radial depths around the roadway.

To ensure the fidelity of the two-dimensional similarity simulation in a mining context, the following similarity criteria must be satisfied among the key parameters:

(1) Geometric similarity

$$C_{l}=\frac{l_{1}}{l_{2}}$$ (1.2)

(2) Gravity (Unit Weight) Similarity

$$C_{\gamma }=\frac{\gamma_{1}}{\gamma_{2}}$$ (1.3)

(3) Stress Similarity

$$C_{\sigma }=\frac{\sigma_{1}}{\sigma_{2}}$$ (1.4)

(4) Elastic Modulus Similarity

$$C_{E}=\frac{E_{1}}{E_{2}}$$ (1.5)

(5) Support Strength Similarity

$$C_{F}=\frac{F_{1}}{F_{2}}=C_{l}^{3}\times C_{\gamma }$$ (1.6)

(6) Poisson’s Ratio Similarity

$$C_{\mu }=\frac{\mu_{1}}{\mu_{2}}$$ (1.7)

(7) Internal Friction Angle Similarity

$$C_{\varphi }=\frac{\varphi_{1}}{\varphi_{2}}$$ (1.8)

To ensure the similarity and validity of the two-dimensional physical model tests for deep mining roadways, the above similarity ratios must satisfy the following comprehensive relationship:


$$C_{\sigma }=C_{\gamma }\times C_{l},\;C_{\sigma }=C_{E},\;C_{\mu }=1,\;C_{\varphi }=1$$ (1.9)

The stress similarity ratio is defined as the product of the unit weight similarity ratio and the geometric similarity ratio. Furthermore, the stress similarity ratio is required to be equal to the elastic modulus similarity ratio. The similarity ratios for both Poisson’s ratio and internal friction angle are set to 1.

In similarity model testing, initial stress similarity refers to the requirement that the internal stress distribution of the model prior to excavation or loading matches the in-situ stress state of the prototype rock mass. This is achieved by adjusting the pressure in hydraulic cylinders to replicate the initial stress field, thereby ensuring that the model can realistically reproduce the stress redistribution and deformation process caused by external loads or excavation activities. Boundary condition similarity ensures that the force and displacement constraints at the model boundaries are consistent with those of the actual engineering conditions. It is also essential to properly design the distance between the tunnel and the model boundaries to minimize boundary effects that may influence the displacement and stress distribution of the surrounding rock.

This study primarily focuses on evaluating the effectiveness of support structures in controlling the creep deformation of surrounding rock. Therefore, selecting model materials that accurately represent the mechanical behavior of the actual support components is of critical importance. According to the support scheme used in the investigated roadway, the main support elements include concrete-filled steel tube (CFST) arches, grouted concrete, casings, struts between arches, steel mesh, rock bolts, and anchor plates.

As described in the previous section, the similarity ratio for the support reaction force is 79,101.56. The selection of model materials for support components must satisfy both mechanical similarity and geometric similarity requirements. Based on previous experimental work conducted by the research group, solder wire has been proven to reliably simulate rock bolts [33]. Tensile tests of solder wires with various diameters were conducted, and 0.42 mm diameter wire was selected as the most suitable analog material. Thin iron sheets were used to simulate the anchor plates, and nuts and bolts were employed to represent the bolt ends, allowing pretension to be applied by tightening the nuts. The anchored sections were fixed in place using transverse iron sheets. The parameters of the model support components are summarized in Table 1.

**Table 1:** Parameters of the support components.

Supporting materials	Category	Length/mm	Diameter/mm	Breaking load/N
Bolt	Actual anchor bolt	2000	20	180000
	simulated material	80	0.42	2.27

**Fig 5:**
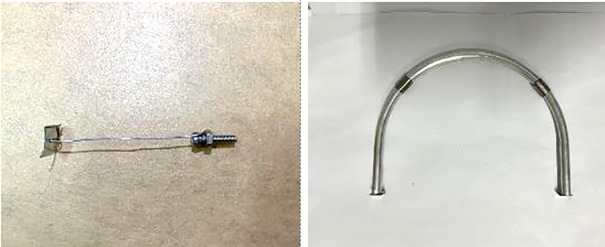
Simulated materials for the support structure components.

The concrete-filled steel tube (CFST) support is designed as a straight-wall semi-circular arch with an outer diameter of 194 mm and a wall thickness of 8 mm. Based on the calculated support force similarity ratio, thin aluminum tubes with a diameter of 7.8 mm and a wall thickness of 0.3 mm were selected as the analog material to simulate the steel tubes. Casings and struts between the arches were also modeled using aluminum tubes. The base of each arch was simulated using square iron sheets, which were bonded to the bottom of the aluminum tubes with AB epoxy adhesive to enhance structural stability. The reinforcement mesh positioned behind the arch supports was simulated using fine aluminum wire mesh. The spacing between adjacent support arches was set to 32 mm, and a total of five arches were installed. The similarity model of the support components is shown in Table 2 and Fig 5.

**Table 2:** Parameters of the Similar Materials for the Support Arches.

Supporting materials	Category	Roadway size/mm	Support diameter/mm	Thickness/mm	Bearing capacity of short columns	Support spacing/mm
Concrete-filled steel tube support	Prototype support	4200 × 3200 mm	194	8	4543kN	800
	Similar simulation materials	168 × 128 mm	7.8	0.3	57.4N	32

In this section, two sets of similarity model tests were designed to comparatively analyze the stress and deformation behavior of surrounding rock under creep loading, with and without support systems. The first group represents a roadway model without any support components. In this test, the surrounding rock remains unsupported, allowing observation of the progressive evolution of internal stress and deformation under long-term creep loading. Radial displacement of the surrounding rock is monitored to analyze its time-dependent displacement behavior, revealing deformation characteristics such as compression and expansion. The stress distribution patterns within the surrounding rock under sustained creep loading are examined, along with failure modes at different radial depths and spatial locations.

The second group consists of a roadway model equipped with a composite support system based on concrete-filled steel tube (CFST) arches. In this test, the surrounding rock is supported by the CFST-based system. By comparing the results of the unsupported and supported models, the effectiveness of the composite support in controlling radial displacement is evaluated. The interaction between the surrounding rock and the support system under creep loading is further analyzed by integrating observations of stress evolution, displacement behavior, and support deformation characteristics.

### 4.2 Two-dimensional mining model test system

**Fig 6:**
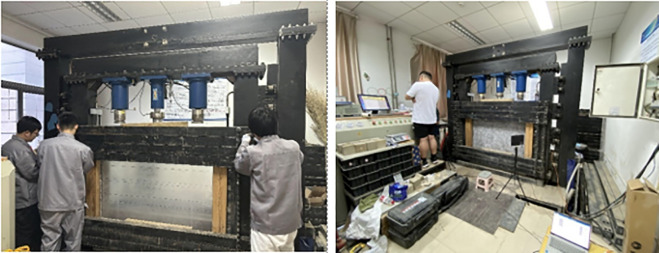
Similarity model test apparatus.

The similarity model test system consists of a loading system, a servo control system, and a displacement monitoring system. It is designed to simulate the mechanical response and deformation behavior of roadway surrounding rock under different support conditions. The model frame has dimensions of 1400 mm in width, 1300 mm in height, and 20 mm in thickness, as shown in Fig 6.

During the test, bidirectional loading is applied to the top and both sides of the model frame using hydraulic cylinders, enabling multi-directional stress simulation. The loading system includes three hydraulic cylinders mounted at the top to apply vertical loads, and 11 hydraulic cylinders installed on each lateral side to apply horizontal loads. The maximum stroke of the top hydraulic cylinders is 250 mm. On each side, two of the lateral cylinders are static and maintain a constant pressure to simulate the static stress field of the surrounding strata, while the centrally positioned cylinder is dynamic and capable of applying cyclic or variable loads, simulating dynamic stress conditions such as those induced by seismic activity or equipment vibrations.

The horizontal hydraulic cylinders have a maximum stroke of 100 mm, allowing controlled application of lateral pressure to simulate in-situ horizontal stress in the rock mass. Mechanical sensors are installed on both the top and lateral hydraulic cylinders to continuously monitor the magnitude and variation of applied forces in real time.

### 4.3 Configuration of support components

In this study, the support components, including rock bolts and wire mesh, were embedded into the model in advance. Based on the predetermined layout, the embedding positions were marked on the front transparent acrylic panel before model construction. During the model filling process, the placement progress was carefully monitored. Once the filling reached the marked positions, the support mesh and bolts were fixed and embedded into the model.

**Fig 7:**
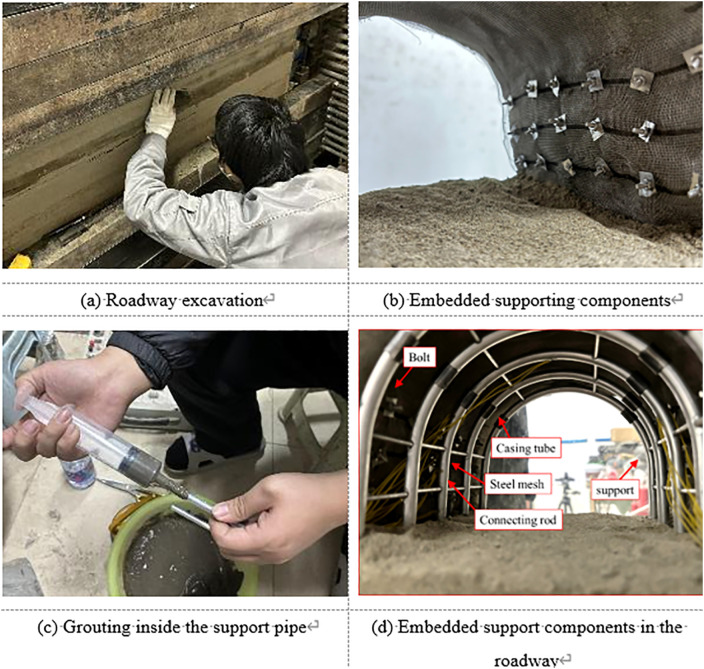
Configuration of support components.

The concrete-filled steel tube (CFST) support structures were installed after the excavation stage was simulated. The CFST arch, designed as a straight-wall semi-circular shape, was composed of two vertical wall segments and a top arch segment, all connected by aluminum sleeves. The core concrete inside the aluminum tubes was simulated using a 1:1 mixture of cement and gypsum. The unset slurry was injected into the tubes using syringes and left to cure for two days, as illustrated in Fig 7.

### 4.4 Experimental monitoring

**Fig 8:**
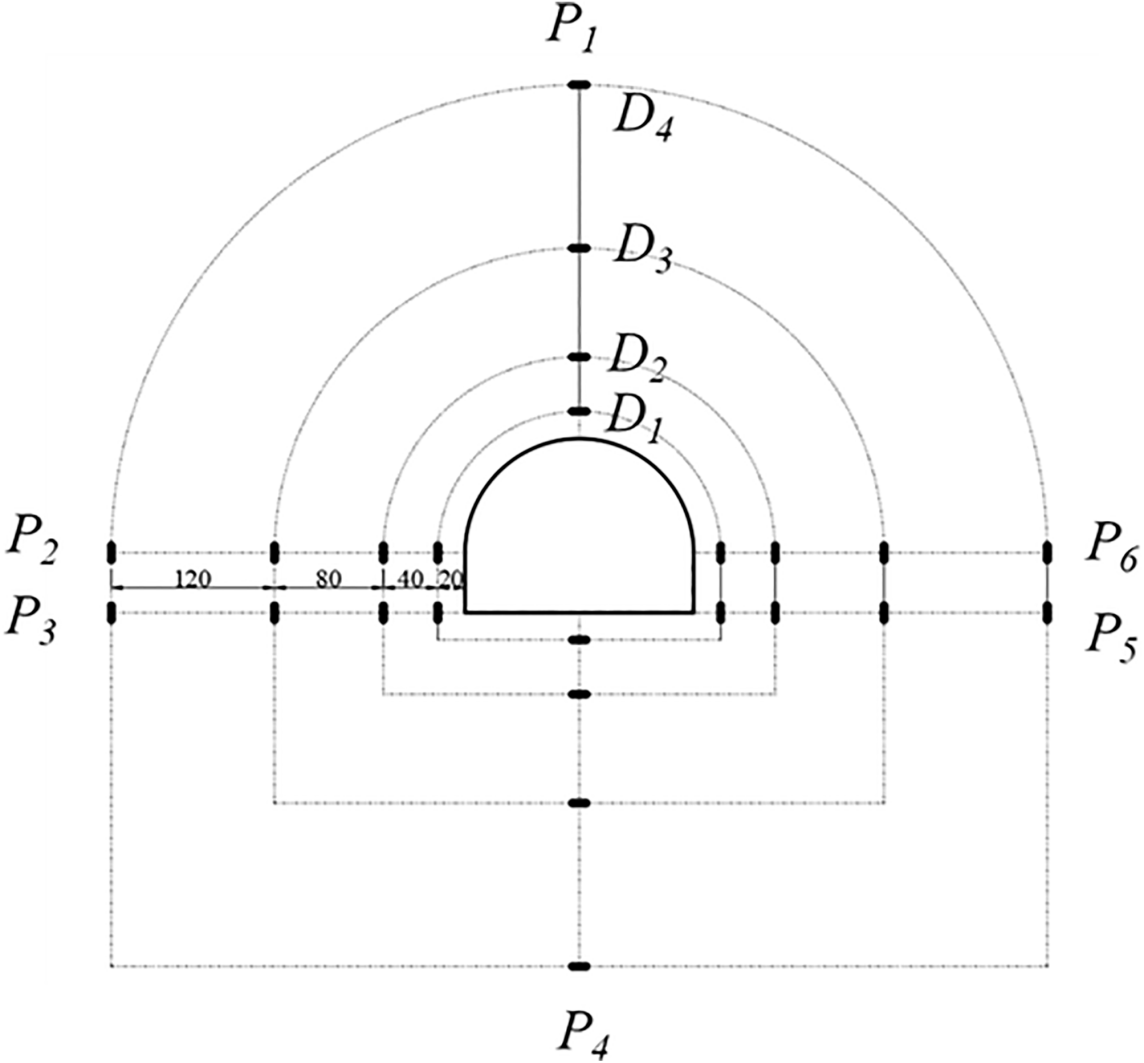
Distribution of deformation data monitoring points.

**Fig 9:**
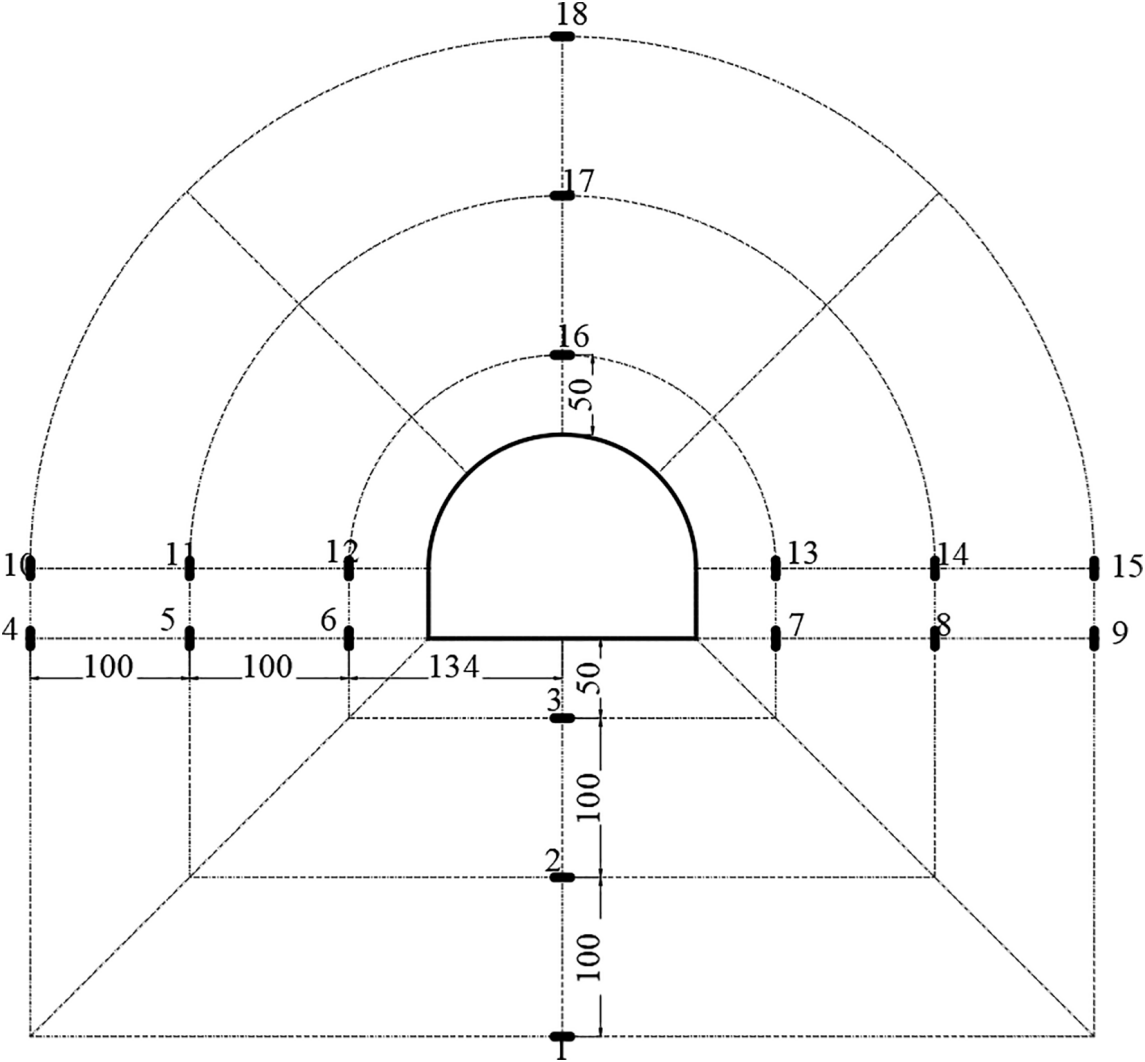
Distribution of stress monitoring points in the model test.

The layout diagram of the displacement monitoring points for the test is shown in Fig 8. *P*_*1*_ to *P*_*6*_ respectively represent the arch top, the left and right arch feet, and the arch base. *D*_*1*_-*D*_*4*_ represent the measurement points arranged outward along the radial depth at each position. A total of 18 stress monitoring points were arranged in the model, as shown in Fig 9. Three vertical monitoring points were positioned below the tunnel floor, starting 50 mm from the bottom and spaced at 100 mm intervals. Similarly, three monitoring points were arranged above the tunnel roof, starting 50 mm from the top and also spaced at 100 mm intervals. Monitoring points 1–3 and 15–18 were used to measure the vertical stress in the roof and floor of the roadway. In addition, 12 monitoring points were installed along the left and right sidewalls (ribs) of the tunnel to measure horizontal stress. The nearest points on each side were located 134 mm horizontally from the tunnel centerline, and subsequent points were arranged outward with a spacing of 100 mm. Monitoring points 4–15 recorded the horizontal stress distribution in the two sidewalls.

Vertical loads were applied to the model using three hydraulic cylinders positioned at the top, while horizontal loading was provided by lateral hydraulic cylinders. The bottom of the model was rigidly constrained. In the longitudinal direction, constraints were applied through the front acrylic panel and a rear steel beam fixed along the model’s front and back edges. The loading scheme is summarized in Table 3.

**Table 3:** Graded Loading Scheme for the Model Test.

Load level/m	Prototype vertical stress/MPa	Prototype horizontal stress/MPa	Model vertical stress/MPa	Model horizontal stress/MPa	Loading at the top of the model/kN	Lateral loading force of the model/kN
*σ*_*0*_	27.35	28.3	0.729	0.755	204.12	196.3
1.2*σ*_*0*_	32.82	33.96	0.875	0.906	245	235.56
1.4*σ*_*0*_	38.29	39.62	1.021	1.057	285.88	274.82
1.6*σ*_*0*_	43.76	45.28	1.167	1.207	326.76	313.82
1.8*σ*_*0*_	49.23	50.94	1.313	1.358	367.64	353.08

First, the initial in-situ stress field *σ*_*0*_ was applied to the model according to the calculated stress similarity ratio. Vertical and horizontal loads were set to 0.729 MPa and 0.755 MPa, respectively. These loads were applied simultaneously and maintained under constant pressure for 20 minutes to simulate the initial stress equilibrium. After this stabilization phase, tunnel excavation was simulated. Upon completion of the excavation, the loading level was maintained for 24 hours, followed by incremental loading steps. Each subsequent loading stage lasted for 24 hours.

After model casting and curing were completed, graded loading was applied to the model. Prior to loading, high-speed cameras were used to capture images of the speckle patterns on the model surface to establish the initial reference plane. After each loading step was stabilized for 10 minutes, another set of images was taken. By comparing the pre- and post-loading images, the displacement field of the surrounding rock was obtained. Before each loading step, all strain gauge acquisition channels were zeroed and automatically balanced. Data collection was initiated after load stabilization at each level. Displacement and stress data of the surrounding rock were recorded at every loading stage. The collected experimental data were subsequently processed and analyzed.

### 4.5 Test results and analysis of the unsupported roadway

#### 4.5.1 Deformation and failure characteristics of the surrounding rock.

**Fig 10:**
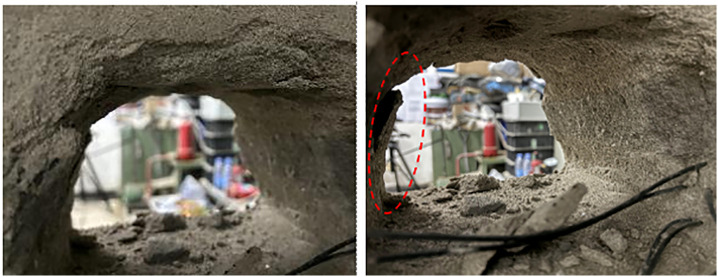
Deformation and failure characteristics of the unsupported roadway.

As shown in Fig 10, following tunnel excavation and the stepwise application of graded loads, the surrounding rock exhibited significant deformation and failure characteristics. From a macroscopic perspective, the entire tunnel cross-section experienced contraction. Local spalling was observed near the edges of the roof arch, indicating either stress concentration or localized degradation of rock strength in this area. Both sidewalls showed clear inward convergence, and the presence of spalling and detachment along the ribs suggests a substantial reduction in structural stability of the surrounding rock on the sidewalls.

#### 4.5.2 Evolution of the displacement field in the surrounding rock.

Displacement images of the tunnel, captured by the high-speed camera at different time intervals, were imported into post-processing software for analysis. Displacement data corresponding to the monitoring point locations were extracted at each time step. These data were then used to plot time–displacement curves, revealing the temporal evolution of deformation at various positions within the surrounding rock. Overall, the displacement at different locations exhibited a slow and continuous increase over time, indicating a pronounced creep effect.

**Fig 11:**
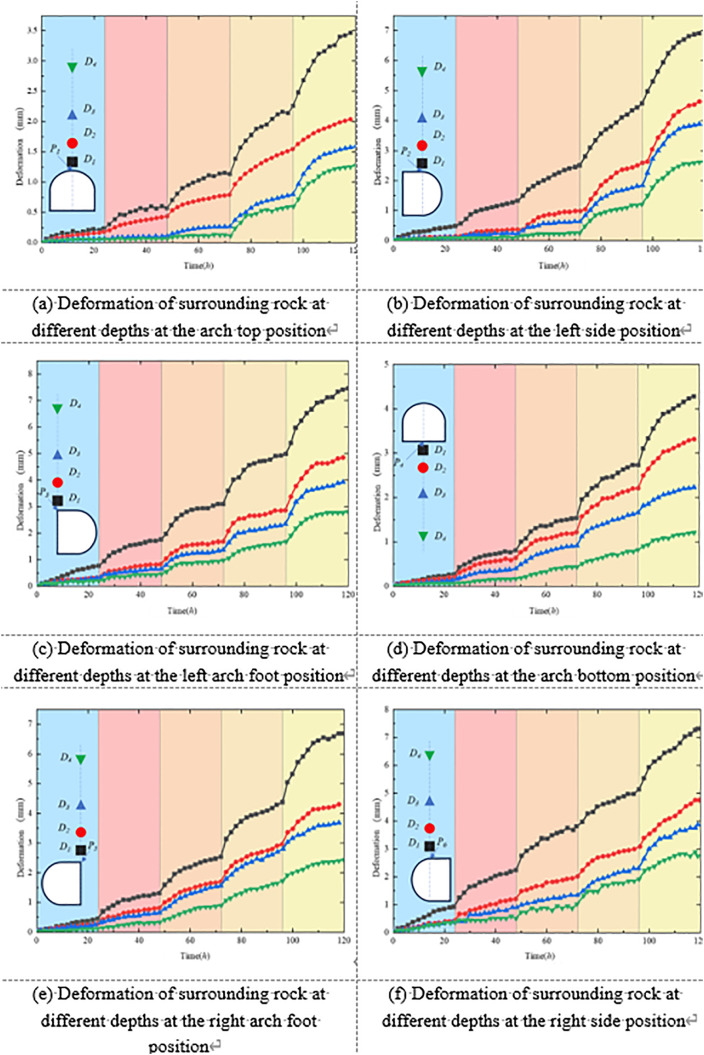
Shows the development of surrounding rock deformation in roadways without support.

Fig 11 illustrates the displacement development trends at typical locations within the surrounding rock at various radial depths under unsupported conditions. In general, the displacement increased progressively with loading duration, reflecting clear time-dependent creep behavior. This trend was especially prominent in stress-concentrated regions such as the roof arch, sidewalls, and haunches. At these locations, shallow surrounding rock exhibited significantly larger displacements than deeper zones, indicating that the rock mass near the excavation boundary experienced more intense stress disturbance and exhibited more sensitive deformation responses. For example, at the roof arch, the shallow monitoring point D1 recorded a maximum radial displacement of 3.46 mm, which is approximately 2.71 times greater than that of the deeper point D4 (1.276 mm). This highlights the attenuation of creep deformation with increasing radial depth in the surrounding rock.

At the sidewalls of the roadway, lateral convergence deformation was particularly pronounced. The maximum horizontal displacements at point D1 on the left and right sidewalls reached 6.90 mm and 7.309 mm, respectively. With increasing depth, the displacement decreased rapidly, falling to 2.62 mm and 2.74 mm at point D4 on the left and right sides, respectively—corresponding to an average reduction in deformation rate of over 60%. A similar trend was observed at the haunches, where the maximum horizontal displacements at point D1 were 7.45 mm (left) and 6.68 mm (right), while displacements at D4 dropped to less than 3 mm. This distribution pattern—from shallow to deep—indicates a clear deformation gradient within the surrounding rock under creep loading. The sidewalls and haunches serve as the primary zones of convergence deformation and exhibit lower stability, which should be emphasized in future support design.

Although the roof–floor junction (invert) exhibited smaller overall deformation compared to other regions, it still demonstrated a significant depth-dependent attenuation. The maximum radial displacement at point D1 in the invert region was 4.275 mm, decreasing to 1.21 mm at D4—representing a 71.7% reduction in deformation amplitude. Taken together, these findings indicate that under unsupported conditions, the surrounding rock of the roadway exhibits strong time-dependent and spatially heterogeneous deformation behavior. The shallow surrounding rock is highly sensitive to creep, with severe deformation, while the deeper rock responds more slowly and to a lesser extent.

Furthermore, under unsupported conditions, the roof displacement curve exhibits a typical stepwise response pattern. Specifically, after the first loading stage, the roof displacement stabilizes rapidly within approximately 6 hours. However, as the loading level increases, the progressive propagation of fractures and accumulation of damage within the surrounding rock cause the stabilization time to extend step by step: about 10 hours for the second stage, 12–15 hours for the third and fourth stages, and, under the fifth stage, displacement remains difficult to stabilize over a prolonged period. At lower stress levels, roof creep is primarily governed by elastoplastic deformation, enabling the surrounding rock to release stress more quickly and achieve convergence. In contrast, at higher stress levels, the effects of fracture propagation and cumulative damage become more pronounced, resulting in markedly delayed stabilization of creep deformation, ultimately manifested as prolonged or even unattainable stabilization.

#### 4.5.3 Evolution of the stress field in the surrounding rock.

**Fig 12:**
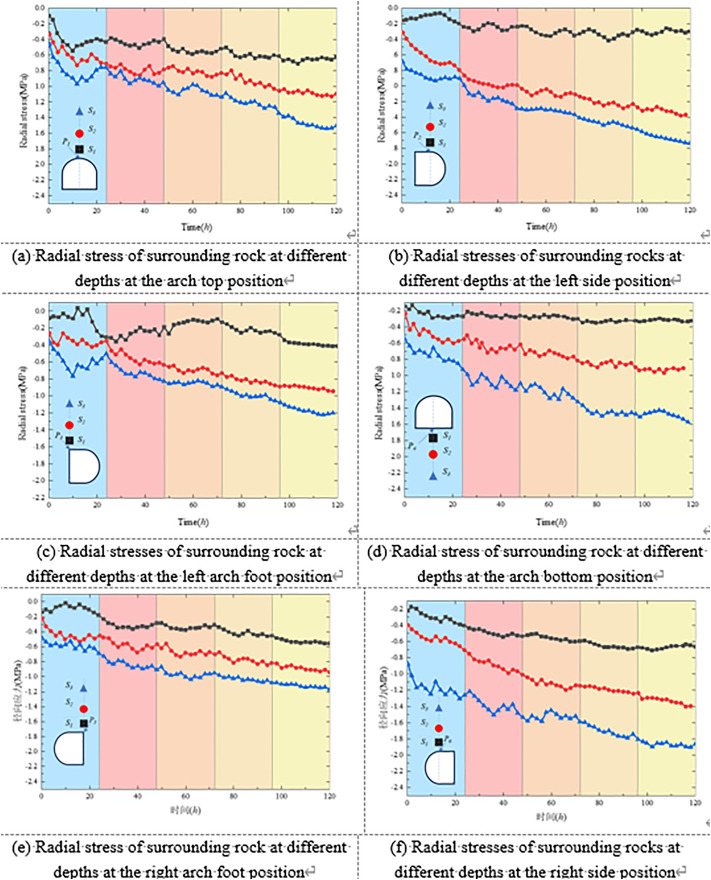
Stress variation law of surrounding rock without support.

Fig 12 illustrates the variation of radial stress in the surrounding rock at different depths and locations under unsupported conditions. Monitoring points were arranged at the roof crown, sidewalls, arch shoulders, and floor, extending from shallow positions (*D*_*1*_/*S*_*1*_) to deeper positions (*D*_*4*_/*S*4), as shown in Figs 8 and 9. Each curve corresponds to a specific monitoring point.

Fig 12 presents the stress evolution characteristics of the surrounding rock at various radial depths in the roof, floor, haunches, and sidewalls under unsupported conditions. Overall, all monitored radial stress values exhibited clear time-dependent behavior. In the initial loading stage, different regions of the surrounding rock experienced a typical stress evolution pattern of “sharp drop–fluctuating recovery” due to excavation-induced disturbance and stress release. Taking the roof as an example, the radial stress at shallow monitoring points dropped rapidly following excavation, then gradually recovered over time, accompanied by slight dynamic fluctuations. In contrast, deeper points showed relatively stable and slowly increasing stress levels, indicating a layered and phased stress transfer mechanism in the roof region.

The sidewall regions exhibited more complex stress evolution processes. As shown in Figs 12b and 12f, at point S1—located nearest to the sidewall boundary—a pronounced radial stress drop occurred during the early excavation stage. In some time intervals, tensile stresses were even observed, suggesting that shallow rock masses underwent intense tensile stress release under unloading. As loading continued, the stress at S1 gradually recovered but remained highly fluctuating. In contrast, deeper points S2 and S3 displayed a smoother, more consistent increase in compressive stress, indicating that the deep surrounding rock remained predominantly in a compressive stress state. A similar trend was observed on the right sidewall, where initial tensile stress also developed and later transitioned into compressive stress. However, the onset of tensile stress occurred earlier than on the left side, indicating a degree of asymmetry in the unloading response between the two sides of the tunnel.

The stress evolution in the haunch and invert (floor center) regions also exhibited clear spatial heterogeneity. In the haunch area, the shallow monitoring points initially showed a brief increase in radial stress during early loading, followed by a rapid decline into tensile stress. As the loading progressed, the stress gradually transitioned back into a compressive state. In contrast, deeper monitoring points in the haunch area exhibited a more stable and progressive increase in stress, indicating mechanical continuity and gradual stress accumulation with depth. At the invert, stress fluctuations were relatively mild overall; however, tensile stress was also observed immediately after excavation, reflecting early-stage stress release in the floor rock mass.

In general, under unsupported conditions, the surrounding rock exhibited a distinct “edge unloading–deep accumulation” pattern in stress field evolution. Different locations showed characteristics such as tensile-to-compressive stress transitions, fluctuating stress responses, and time-dependent stress accumulation.

### 4.6 Test results and analysis of the composite-supported roadway

#### 4.6.1 Deformation and failure characteristics of the surrounding rock.

**Fig 13:**
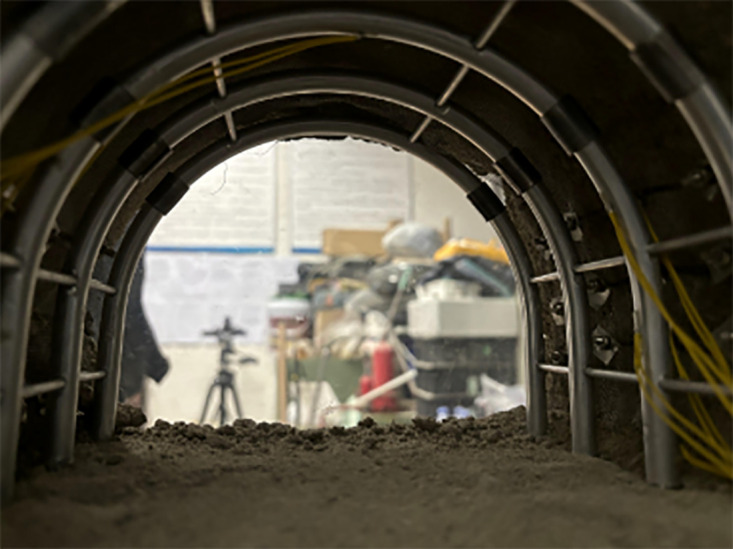
Support scheme of concrete-filled steel tube support.

The model test results for the composite support scheme using concrete-filled steel tube (CFST) arches are shown in Fig 13. The results demonstrate that this support configuration significantly improves the stability of the surrounding rock. As a composite structure, the CFST arch provides efficient radial support force. Compared to the unsupported condition, it offers clear advantages in both support performance and structural stability.

Specifically, the CFST system enhances the lateral confinement of the surrounding rock near the tunnel boundary, effectively reducing deformation-induced structural damage and improving the overall load-bearing capacity of the rock mass.

Under identical loading conditions, the test results show that the composite support system significantly reduced cross-sectional convergence of the tunnel. The high load-bearing capacity of the CFST arches increased confinement on the surrounding rock, resulting in more uniform stress transfer and a notable reduction in overall deformation. However, some localized spalling and detachment still occurred in the sidewalls. Although these failures were considerably mitigated compared to the unsupported case, they indicate that stress concentration in the sidewall region remains an issue that warrants attention. The support structure was especially effective in controlling deformation at the tunnel crown. The CFST arches successfully restrained damage and displacement in this critical area, demonstrating strong support performance and reducing the risk of local failure in the surrounding rock.

#### 4.6.2 Evolution of the displacement field in the surrounding rock.

**Fig 14:**
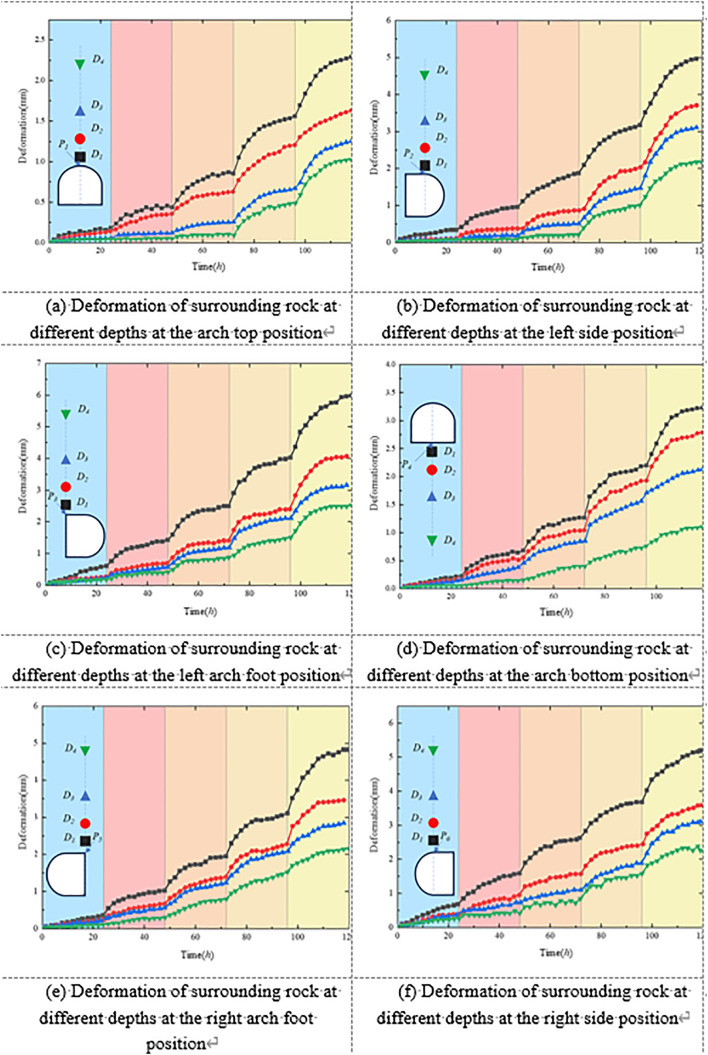
Shows the development of surrounding rock deformation in the roadway under the combined support scheme.

Fig 14 illustrates the displacement evolution of the surrounding rock at typical tunnel locations and various radial depths under the composite support condition using concrete-filled steel tube (CFST) arches. Overall, compared with the unsupported condition, the composite support system significantly reduced displacement magnitudes at all monitoring points and effectively suppressed the rate of deformation development. This indicates that the system possesses strong confinement capacity and excellent deformation resistance.

Taking the roof arch area as an example, the maximum radial displacements at monitoring points D1 to D4 were 2.29 mm, 1.63 mm, 1.27 mm, and 1.05 mm, respectively. The corresponding average deformation rates decreased progressively from 0.019 mm/h to 0.008 mm/h, all of which are notably lower than those recorded at the same positions in the unsupported test. These results demonstrate a significant improvement in the overall stability of the surrounding rock at the tunnel crown under the action of composite support.

From the deformation response of the sidewalls, the composite support system effectively constrained the convergence of the surrounding rock. The radial displacements at point D1 on the left and right sidewalls were 4.96 mm and 5.19 mm, respectively, representing a reduction of approximately 28%–29% compared to the unsupported condition. At the deeper monitoring point D4, the displacements decreased further to 2.17 mm and 2.26 mm, indicating that the suppressive effect of the composite support became more pronounced with increasing depth. In terms of deformation rate, the reduction was especially significant at shallow depths, with average decreases exceeding 55%. This suggests that the CFST arches provided continuous and high-stiffness confinement, effectively slowing the plastic deformation tendency of the shallow sidewall rock under the coupled influence of high in-situ stress and creep.

In addition, the displacements at the haunch and invert regions were also substantially reduced compared to the unsupported case. The horizontal displacements at point D1 on the left and right haunches were 5.98 mm and 4.83 mm, representing reductions of approximately 20%–28%, indicating that the support structure provided effective load transfer and shear resistance in these areas. For the invert region, the radial displacements from D1 to D4 were 3.23 mm, 2.79 mm, 2.14 mm, and 1.10 mm, respectively, with the maximum displacement and rate reductions exceeding 65%. This further confirms the effectiveness of the composite support system in mitigating vertical uplift of the surrounding rock.

Overall, the results shown in Fig 14 demonstrate that the composite support system significantly improves the deformation response of the surrounding rock throughout the tunnel cross-section, particularly in shallow zones with high stress disturbances.

#### 4.6.3 Evolution of the stress field in the surrounding rock.

**Fig 15:**
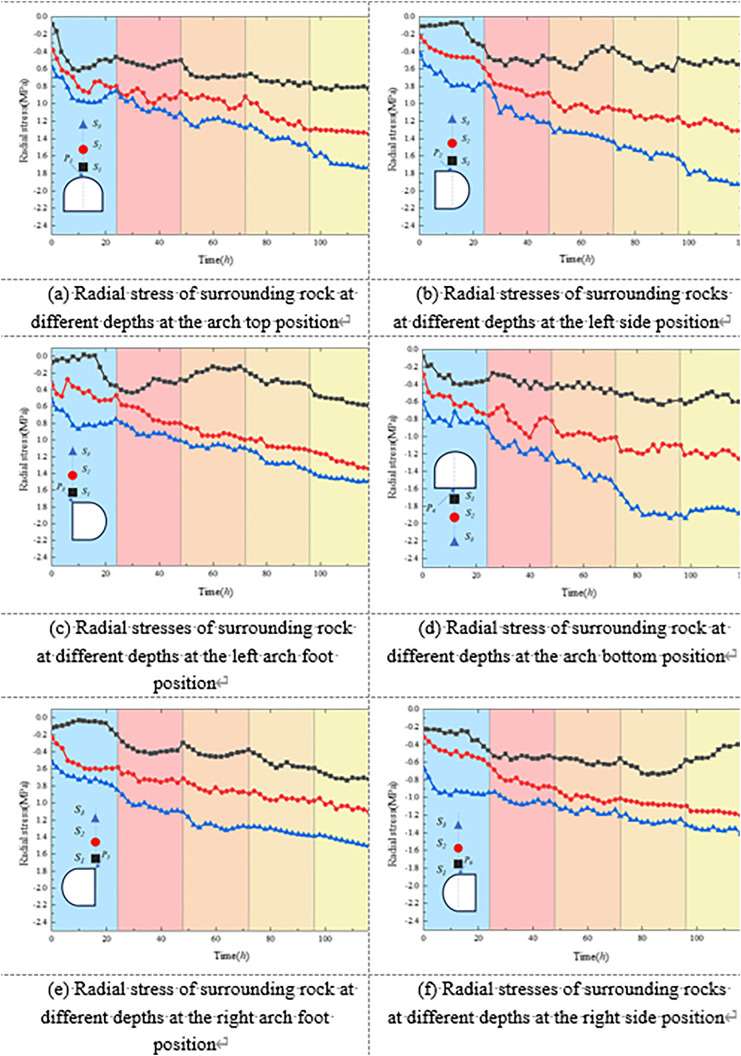
Stress evolution law of surrounding rock under the combined support scheme.

Fig 15 illustrates the temporal evolution of stress in the surrounding rock under the composite support condition. Overall, the concrete-filled steel tube (CFST) support system effectively alleviated the sharp stress drops and tensile stress concentrations in the shallow zones induced by excavation, thereby significantly improving the stress environment of the surrounding rock.

In the roof and floor regions, the monitored stresses generally exhibited a fluctuating upward trend over time, indicating that the support structure provided consistent confinement, enabling a smoother transition of stress release in shallow disturbed zones. Although stress reduction and even transient tensile stress were still observed at shallow monitoring points in the sidewalls and haunches during early excavation, the recovery rate was notably faster than that observed under unsupported conditions. Moreover, the stress increase in the middle and deep surrounding rock was more stable, reflecting the enhanced support effectiveness of the composite system.

Compared with the unsupported case, the composite support substantially improved the continuity of stress transfer and the overall stability of the surrounding rock. The support system promoted a more rational stress redistribution, effectively suppressing shallow stress disturbances while allowing deeper rock masses to better mobilize their load-bearing capacity. In particular, the pronounced stress asymmetry previously observed in the sidewalls was significantly mitigated. No severe tensile stress concentrations reappeared in critical regions such as the haunches and sidewalls, suggesting that the composite support system exhibits excellent unloading-buffering and mechanical coordination capabilities.

## 5 Numerical simulation of creep control techniques in deep roadways

### 5.1 Development of the numerical model

Due to the considerable burial depth of the pump station roadway in the a mine in North China, the surrounding rock undergoes significant creep deformation under prolonged high in-situ stress, which has necessitated multiple rounds of repair. Repeated disturbance from these repairs has progressively weakened the structural integrity of the surrounding rock, making the original support scheme ineffective in controlling its creep deformation and failure. To further investigate the deformation mechanisms of the surrounding rock and evaluate the effectiveness of different support schemes, a three-dimensional numerical model was developed using FLAC3D software, based on the actual geological conditions of the pump station roadway. The model was used to systematically study the stress and displacement responses of the surrounding rock under various support conditions.

**Fig 16:**
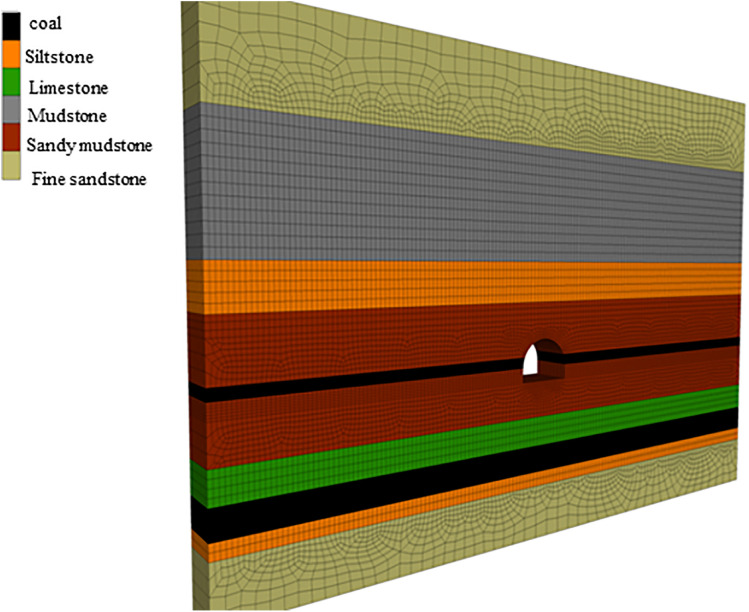
Meshing of the numerical model.

Fig 16 shows an overview of the numerical model. The model dimensions are 50 m in length, 3.2 m in width, and 36 m in height. After meshing, the model comprises a total of 89,432 elements and 101,439 nodes. The boundary conditions and loading scheme were configured to accurately replicate the in-situ stress environment of the actual roadway.

A uniform vertical load of 27.35 MPa was applied to simulate the overburden stress from the overlying strata. A trapezoidal horizontal load of 28.3 MPa was applied to represent the combined effects of in-situ horizontal stress and tectonic stress acting on the coal-bearing rock mass. The measured lateral pressure coefficient was 1.03. Horizontally, the X and Y boundaries were constrained against lateral displacement, while the bottom boundary (Z-direction) was fixed to restrict vertical movement. The mechanical parameters of the surrounding rock used in the numerical model were derived from laboratory tests conducted on rock samples collected from the field site. These parameters are listed in Table 4.

**Table 4:** Parameters of the bracket structure Unit.

Support type	Cross-sectional area (cm^2^)	Elastic modulus (GPa)	Poisonby	Moment of inertia of section	
				*I_x_*(cm^4^)	*I_y_*(cm^4^)
U36	45.69	210	0.27	928.65	1244.75
Steel tube	46.75	206	0.30	2452.30	2452.30
Fill concrete	248.85	41	0.22	4497.25	4497.25

To address the characteristics of surrounding rock subjected to long-term high in-situ stress and creep loading, Liu [34] proposed a damage-based creep constitutive model that accounts for the evolution of damage under creep conditions and incorporates a nonlinear stress–strain relationship. In this study, the proposed constitutive model was compiled as a dynamic link library (DLL) and successfully implemented in FLAC3D via the user-defined constitutive interface.

During the creep process, the viscosity coefficient of the surrounding rock evolves continuously over time. Considering the effect of creep-induced damage, the viscosity coefficient in the accelerated creep stage is expressed as:


$$\eta (t)=\eta_{3}(1-D_{c})$$ (1.10)

Where *Dc* represents the creep damage coefficient and *η*_*3*_ represents the initial viscosity coefficient, then:


$$D_{c}=1-e^{-wt}$$ (1.11)

**Fig 17:**
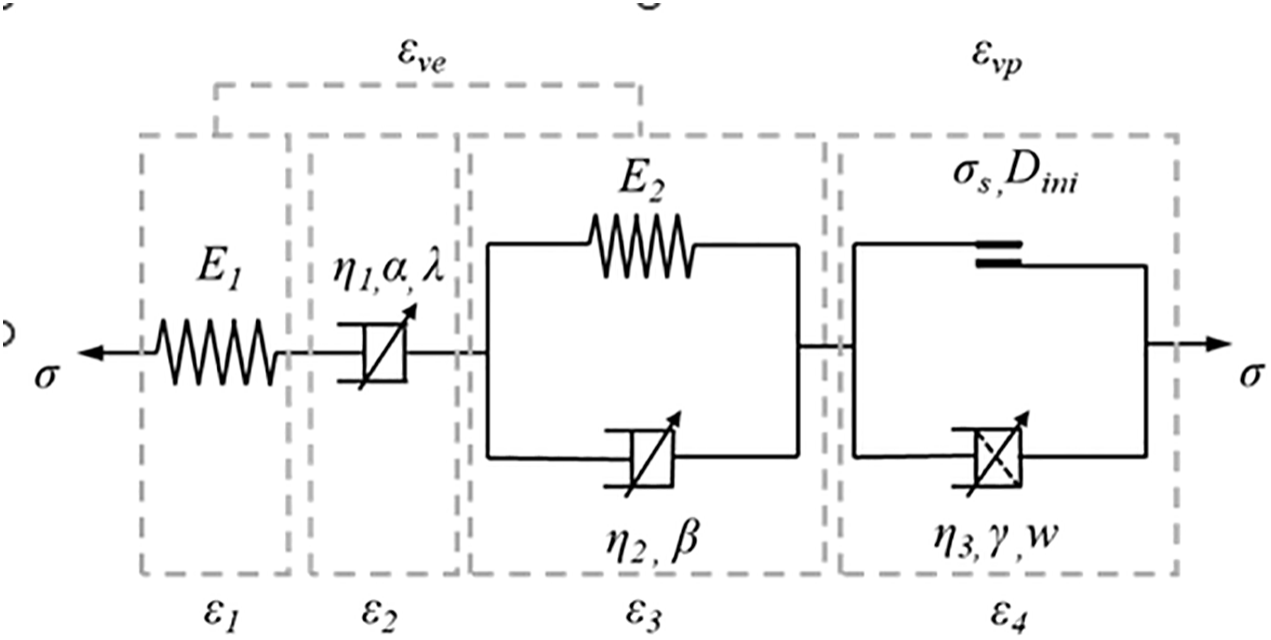
Schematic diagram of the constitutive model of nonlinear creep damage.


The schematic diagram of this constitutive model is shown in Fig 17. Based on the classic Burgers model, the author introduced a fractional-order Abel clay pot to replace the traditional Newton clay pot and designed a viscoplastic body considering creep damage, which was connected in series with it to form a complete nonlinear creep damage model. Its three-dimensional constitutive equation is as follows:

$$\begin{array}{c} \varepsilon_{11}(t)=\frac{\sigma_{1}+2\sigma_{3}}{9K_{0}}+\frac{\sigma_{1}-\sigma_{3}}{3G_{0}}+\frac{\sigma_{1}-\sigma_{3}}{3\eta_{1}e^{-\lambda }}\frac{t^{\alpha }}{\Gamma (1+\alpha )}\mathrm{\backslash vspace}\mathrm{-2mm}\\ \;\;\;\;\;\;\;\;\;\;\;\;\;\;\;\;\;\;\;\;\;\;\;\;\;\;\;\;\;\;\;\;\;\;\;\;\;\;\;\;\;\;\;\;\;\;\;\;\;\;\;\;\;\;\;\;\;\;\;\;\;\;\;\;\;\;\;\;\;\;\;\;\;\;\;\;\;\;\;\;\;\;\;\;\;\;\;\;\;\;\;\;\;\;\;\;\;\;\;\;\;\;\;\;\;\;\;(\sigma_{1}-\sigma_{3}<\sigma_{s})\mathrm{\backslash vspace}\mathrm{-1mm}\\ +\frac{\sigma_{1}-\sigma_{3}}{3G_{1}}-\frac{\sigma_{1}-\sigma_{3}}{3G_{1}}E_{\beta ,1}\left[-G_{1}\frac{t^{\beta }}{\eta_{2}}\right] \end{array}$$ (1.12)

$$\begin{array}{c} \varepsilon_{11}(t)=\frac{\sigma_{1}+2\sigma_{3}}{9K_{0}}+\frac{\sigma_{1}-\sigma_{3}}{3G_{0}}+\frac{\sigma_{1}-\sigma_{3}}{3\eta_{1}e^{-\lambda }}\frac{t^{\alpha }}{\Gamma (1+\alpha )}+\frac{\sigma_{1}-\sigma_{3}}{3G_{1}}\mathrm{\backslash vspace}\mathrm{-2mm}\\ \;\;\;\;\;\;\;\;\;\;\;\;\;\;\;\;\;\;\;\;\;\;\;\;\;\;\;\;\;\;\;\;\;\;\;\;\;\;\;\;\;\;\;\;\;\;\;\;\;\;\;\;\;\;\;\;\;\;\;\;\;\;\;\;\;\;\;\;\;\;\;\;\;\;\;\;\;\;\;\;\;\;\;\;\;\;\;\;\;\;\;\;\;\;\;\;\;\;\;\;\;\;\;\;\;\;\;\;\;\;\;\;\;\;\;\;\;\;\;\;\;\;\;\;\;\;\;\;\;\;\;\;(\sigma_{1}-\sigma_{3}\geq \sigma_{s})\mathrm{\backslash vspace}\mathrm{-1mm}\\ -\frac{\sigma_{1}-\sigma_{3}}{3G_{1}}E_{\beta ,1}\left[-G_{1}\frac{t^{\beta }}{\eta_{2}}\right]+\frac{\sigma_{1}-\sigma_{3}-\sigma_{s}}{6\eta_{3}(1-D_{ini})}t^{\gamma }E_{1,1+\gamma }(wt) \end{array}$$ (1.13)

*E*_*1*_ denotes the elastic modulus of the elastic element, where *α* represents the fractional order, *η*_*1*_ is the viscosity coefficient of the Abel dashpot, and *λ* is a parameter related to the stress level. *E*_*2*_ corresponds to the elastic modulus of the Hookean body, while *η*_*2*_ denotes the viscosity coefficient of its Abel dashpot, with *β* representing the fractional order of the viscoelastic Abel dashpot. *η*_*3*_ is the viscosity coefficient of the dashpot in the viscoplastic component of the rock, and *γ* denotes its fractional order.

The support components in the roadway were modeled using structural elements available in FLAC3D. The U36 steel arch and the concrete-filled steel tube (CFST) arch were simulated using Beam elements, which exhibit mechanical behavior similar to structural beams. This allows for a realistic representation of the support structures’ response under axial force, bending moment, and shear force. The structural parameters of the U36 steel and CFST arches—such as cross-sectional dimensions, elastic modulus, density, and flexural stiffness—were defined based on actual engineering data listed in Table 4 to ensure the model accurately represents their load-bearing capacity and deformation behavior.

Cable elements were used to simulate rock bolts. These elements are designed to capture the mechanical response of slender structural members (e.g., bolts and tendons) under tension, shear, and bending. The input parameters for the rock bolts, including length, diameter, elastic modulus, yield strength, and anchorage properties (e.g., bond strength and anchorage length), were defined according to the data provided in Table 5.

**Table 5:** Parameters of Anchor bolt Structural Units.

Support type	Unit type	Unit type (mm)	Cross-sectional area (cm^2)^	Elastic modulus (Gpa)	Preload (kN)	Tensile strength (kN)
Bolt	Cable	20	3.14	210	100kN	180

The surface support system—comprising wire mesh and shotcrete—was modeled using Shell elements, which are suitable for simulating thin-walled structural components and can effectively capture the mechanical behavior of shell-type support structures. In the model, the wire mesh provides surface constraint to the surrounding rock, while the shotcrete contributes to the overall surface stiffness and integrity of the support system.

### 5.2 Analysis of numerical simulation results

**Fig 18:**
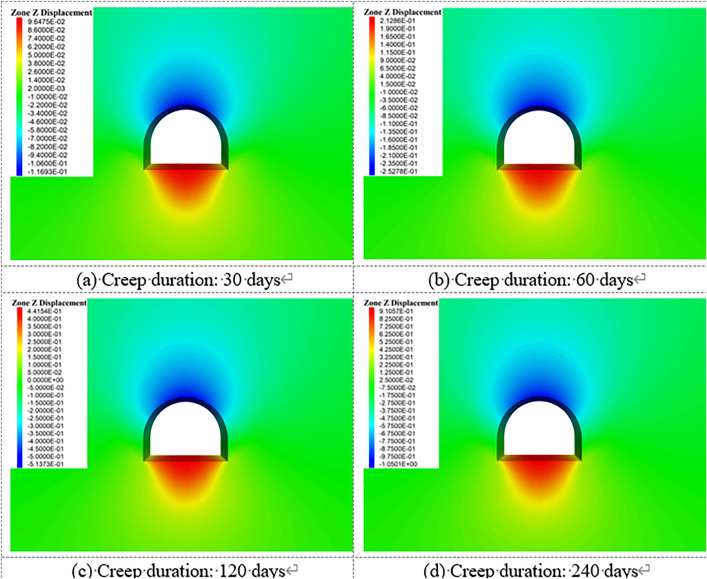
Vertical displacement of surrounding rock.

**Fig 19:**
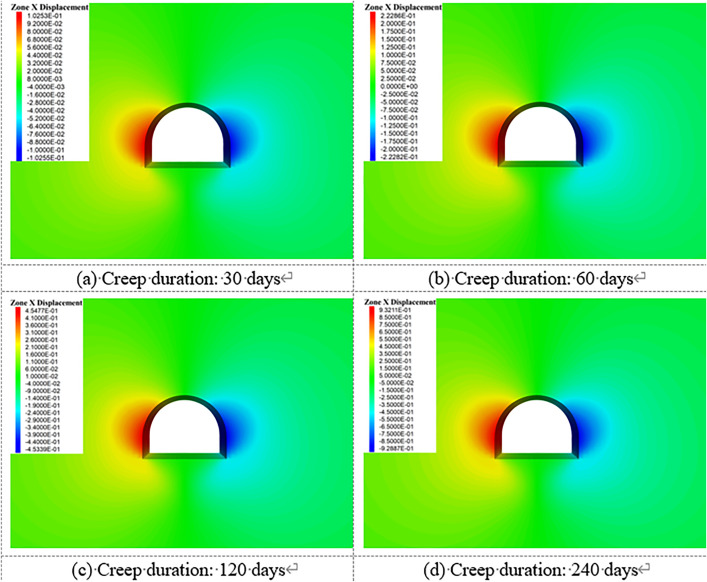
Horizontal displacement of surrounding rock.

**Fig 20:**
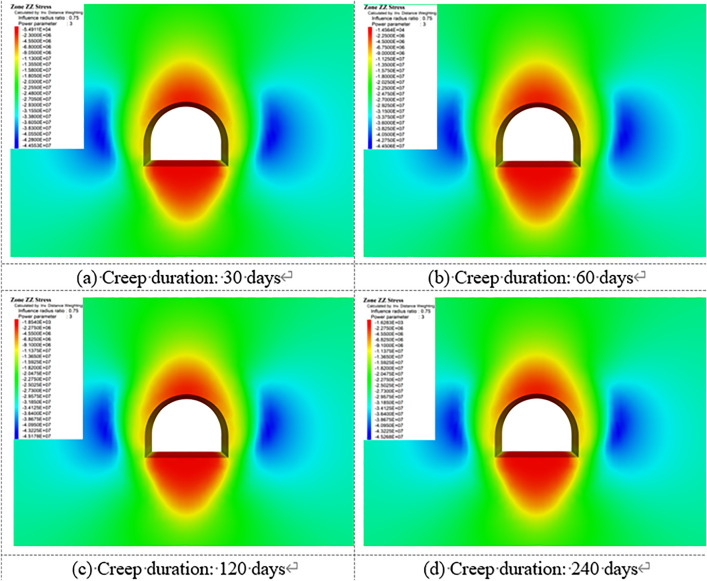
Vertical stress of Surrounding rock.

**Fig 21:**
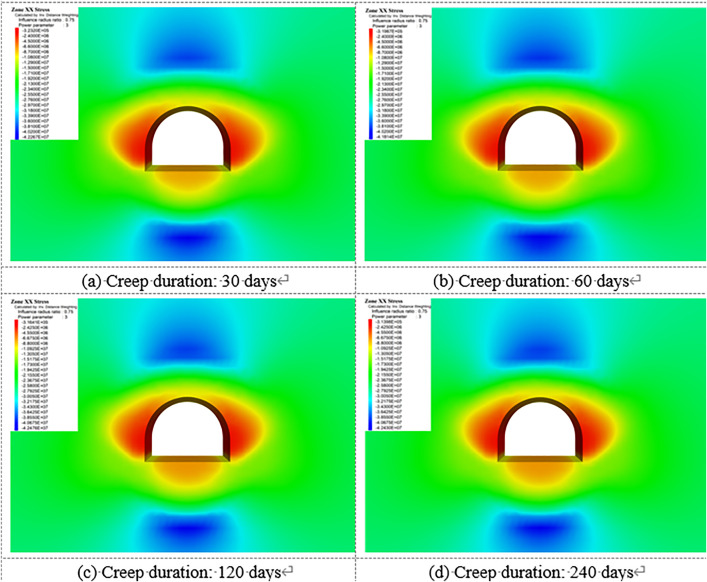
Horizontal stress of surrounding rock.

**Fig 22:**
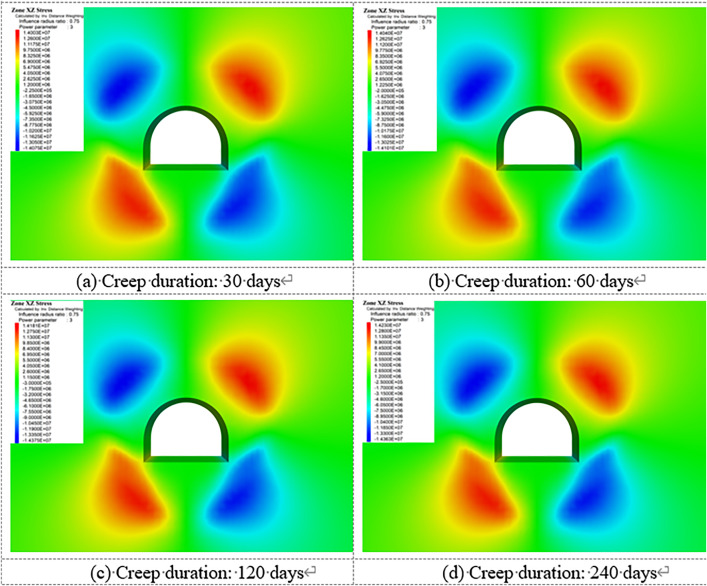
Shear stress of surrounding rock.

Figs 18–22 show the mechanical response characteristics of the surrounding rock under unsupported conditions at creep durations of 30 days, 60 days, 120 days, and 240 days, respectively. The figures reveal substantial changes in vertical displacement, horizontal displacement, and stress field distribution, indicating that the creep effect has a significant impact on roadway stability.

Under the influence of creep, the surrounding rock gradually deforms inward toward the excavation, resulting in increasing vertical displacement over time, manifested as roof subsidence and floor heave. At a creep duration of 30 days, displacements at the roof and floor are relatively minor. However, by 240 days, the roof subsidence reaches 91.1 cm and the floor uplift reaches 105 cm, indicating a substantial increase in deformation.

By comparing the similarity model results in Section 4 with the numerical simulation outcomes, it can be observed that the simulated deformation trends are highly consistent with those measured in the physical tests. Both approaches captured the progressive convergence of the sidewalls and the subsidence of the roof under long-term creep loading. Moreover, the stress distribution patterns obtained from the simulation closely match those recorded in the physical model, particularly in the stress concentration zones near the arch foot and the unloading zones at the roof and floor.

Horizontal displacement is mainly concentrated in the sidewall regions, showing significant convergence of the surrounding rock toward the tunnel interior. As the creep duration extends from 30 to 240 days, the maximum horizontal displacement at the sidewalls increases from 10.3 cm to 93.2 cm—a dramatic rise. This notable increase highlights the progressive weakening of sidewall stability due to long-term creep. In the early stages, horizontal displacement is localized, but over time, the deformation zone expands symmetrically along both sidewalls, forming large-scale displacement regions.

The vertical stress distribution within the surrounding rock varies significantly across different regions. At approximately 2 meters from the tunnel sidewalls, distinct vertical stress concentration zones are observed, with peak values reaching up to 45.3 MPa, indicating substantial compressive loading. Meanwhile, arch-shaped unloading zones appear near the roof and floor, where the stress values are significantly lower than those in the surrounding rock. This indicates partial release of initial stress during the creep process after excavation, leading to stress redistribution in these areas.

The distribution characteristics of horizontal stress are nearly opposite to those of vertical stress. In the sidewall regions, excavation induces significant stress relief, forming symmetrically distributed low-stress zones. In contrast, horizontal stress concentration zones appear approximately 2 m away from the roof and floor, with a peak value reaching 42.5 MPa. This distribution indicates that creep-induced stress redistribution causes horizontal stresses to concentrate in specific areas, while the sidewalls exhibit reduced stress levels due to deformation-driven stress release.

Shear stress is primarily concentrated at the four corners of the roadway, exhibiting pronounced localized stress concentration. At 120 days of creep, the maximum shear stress reaches 14.38 MPa and then gradually stabilizes. This shear stress concentration suggests that the corner regions are prone to mechanical heterogeneity during creep, posing potential instability risks. The distribution of shear stress is closely related to the geometry of the roadway and is influenced by the damage evolution of the surrounding rock under creep conditions.

Creep duration has a significant influence on the displacement and stress distribution of the surrounding rock. As the creep time increases, both vertical and horizontal displacements increase markedly, manifesting as overall convergence deformation of the roadway. Meanwhile, vertical and horizontal stresses exhibit zones of concentration and unloading in specific regions, reflecting the redistribution of stresses induced by creep. Concentrated shear stress zones highlight areas of localized instability within the surrounding rock. These findings demonstrate that, under unsupported conditions, creep significantly exacerbates deformation and stress variation in the surrounding rock. In particular, the accumulation of stress concentration and deformation in critical regions such as the roof, floor, and sidewalls poses a substantial threat to the long-term stability of the roadway.

**Fig 23:**
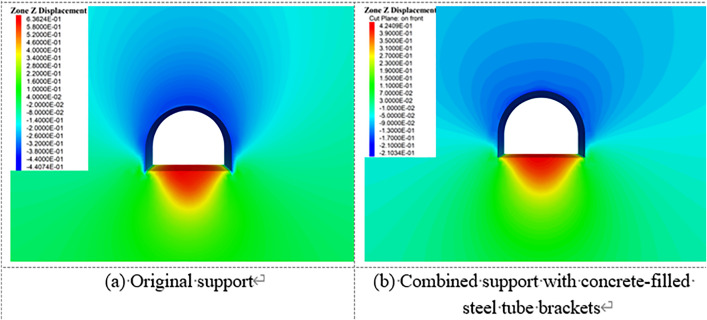
Vertical displacement of surrounding rock.

**Fig 24:**
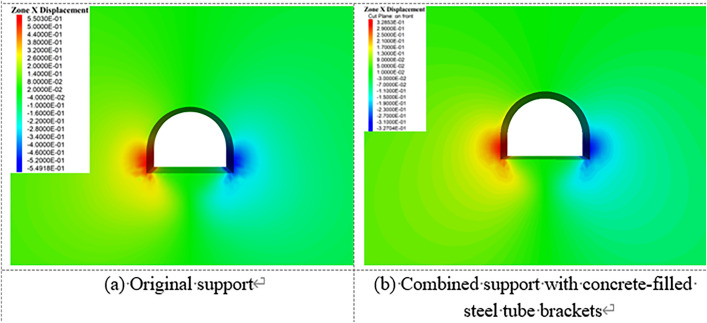
Horizontal displacement of surrounding rock.

**Fig 25:**
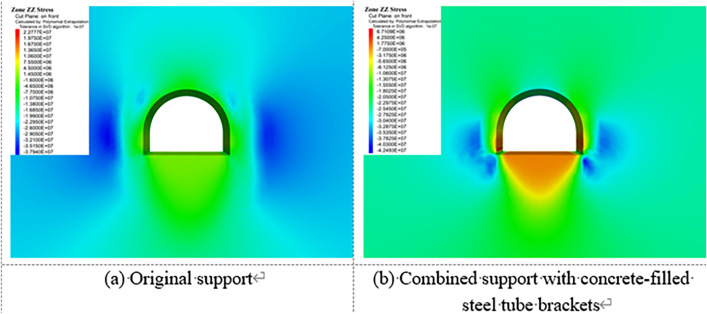
Vertical stress of Surrounding rock.

**Fig 26:**
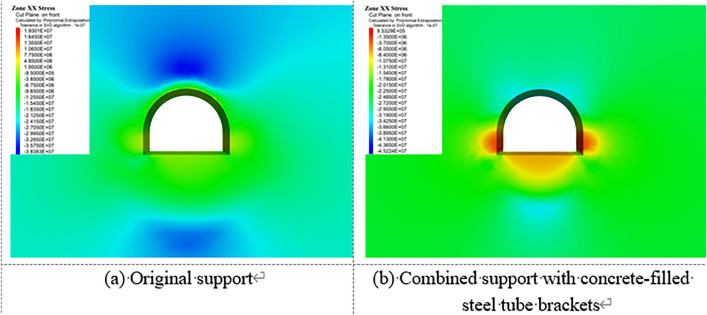
Horizontal stress of surrounding rock.

**Fig 27:**
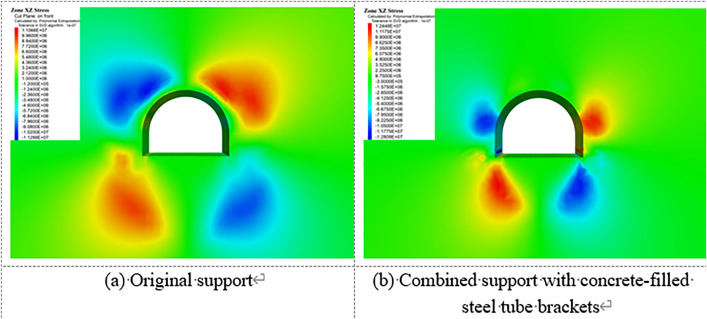
Shear stress of surrounding rock.

Figs 23–27 illustrate the mechanical response of the surrounding rock under both the original support scheme and the composite support scheme utilizing circular concrete-filled steel tube (CFST) arches, with a consistent creep duration of 240 days. It is evident that the joint support system significantly improves the mechanical behavior of the roadway.

Specifically, the maximum vertical displacement at the roof under the joint support is 21 cm, while the maximum floor heave reaches 42.4 cm, representing reductions of 33.3% and 52.4%, respectively, compared to the original support. The maximum horizontal displacement at the sidewalls is 32.8 cm, a 40.3% decrease from the original scheme. Additionally, the extent of the plastic zone in the surrounding rock is notably reduced under the joint support system.

In summary, the new composite support approach not only markedly enhances the structural bearing capacity, thereby controlling the progressive deformation of the roadway, but also improves the physical and mechanical properties of the surrounding rock due to the combined effects of grouting and bolting. This effectively suppresses the development of the plastic zone and enhances the overall stability of the deep roadway under long-term creep loading.

**Fig 28:**
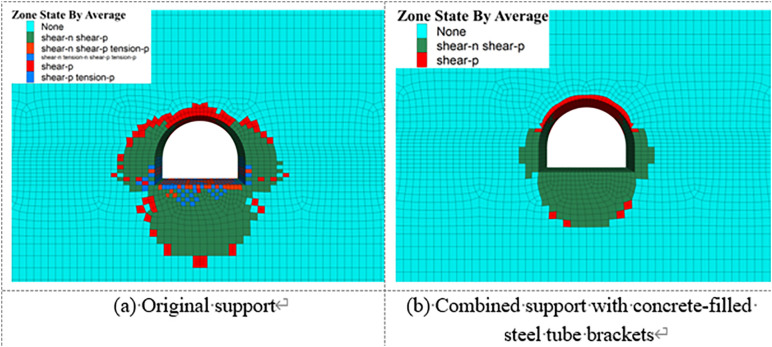
Range of the plastic zone of surrounding rock.

Figs 28 present the distribution of plastic zones in the surrounding rock under both the original support scheme and the composite support scheme using concrete-filled steel tube (CFST) arches. Comparative analysis indicates that, under the original support scheme, the plastic zone in the surrounding rock is significantly larger and mainly concentrated in the floor and sidewall areas. This suggests that the original support system lacks sufficient capacity to control damage in the surrounding rock under high in-situ stress and is ineffective in resisting creep-induced deformation.

In contrast, the plastic zone under the CFST composite support scheme is substantially reduced, with both the extent and the magnitude of plastic strain in concentrated regions markedly lower than those under the original support. Compared with the original scheme, the distribution of the plastic zone becomes more favorable. This improvement is attributed to the stronger confining effect of the composite support system and the synergistic contribution of grouting and rock bolts, which significantly enhance the mechanical properties and overall strength of the surrounding rock. As a result, the propagation of the plastic zone is effectively suppressed.

### 5.3 Engineering applicability analysis

To evaluate the practical feasibility of the proposed composite support system, a comparative analysis was conducted with reference to existing engineering cases. The results indicate that:

(1) Construction process – The concrete-filled steel tube (CFST) support offers convenient assembly and strong adaptability to complex roadway cross-sections, thereby reducing on-site construction disturbances.
(2) Material durability – Concrete infill significantly enhances the steel tube’s resistance to corrosion and creep, extending the service life of the structure.
(3) Maintenance cycle – Compared with traditional U-shaped steel sets, the composite support system reduces the frequency of secondary reinforcement, substantially lowering maintenance demands.
(4) Economic efficiency – Although the initial investment is higher, the system demonstrates superior overall cost-effectiveness when evaluated on a full life-cycle basis.

## 6 Conclusion

(1) The surrounding rock in deep underground roadways, subjected to high in-situ stress and prolonged creep loading, displays pronounced time-dependent deformation and spatial variability. In unsupported conditions, displacement accumulates progressively over time, with shallow zones undergoing significantly greater deformation than deeper regions. This reveals a characteristic attenuation with depth. The arch roof and sidewalls emerge as primary deformation-prone zones, posing critical risks to the structural integrity of the roadway.
(2) The implementation of a steel–concrete composite support system markedly mitigates creep-induced deformation and promotes a more favorable stress redistribution. Compared with the unsupported case, this integrated support approach significantly reduces roof subsidence, sidewall convergence, and floor heave—achieving 30%–60% average reduction in shallow deformation—and ensures effective confinement and stabilization of critical zones.
(3) Experimental findings elucidate a stress evolution mechanism characterized by shallow unloading and deep stress concentration. The composite support system effectively buffers abrupt stress release and promotes gradual stress redistribution. In sidewall and arch-foot regions, it markedly suppresses the development of tensile stresses during the early stage, enhancing the continuity of load-bearing capacity and mechanical synergy within the surrounding rock.
(4) Numerical simulations incorporating the damage-based creep constitutive model show strong agreement with physical experiments, further corroborating the deformation control efficacy of the composite support system. Under long-term service conditions, the system effectively restrains the evolution of plastic zones and decelerates the degradation of surrounding rock, providing a robust theoretical framework and engineering basis for support optimization and long-term stability assessment in deep roadways.

## Supporting information

S1 Data Minimal dataset including raw values underlying all figures and tables. (XLSX)
